# Risk of neurodevelopmental disorders after fetal topiramate exposure: a systematic review

**DOI:** 10.3389/fdsfr.2026.1761506

**Published:** 2026-03-27

**Authors:** Renate Eikevåg Lundøy, Anniken Sigvathsen, Marte-Helene Bjørk, Nils Erik Gilhus, Jenny Linnea Victoria Lindroos

**Affiliations:** 1 Faculty of Medicine, University of Bergen, Bergen, Norway; 2 Department of Clinical Medicine, University of Bergen, Bergen, Norway; 3 Neurological Department, Haukeland University Hospital, Bergen, Norway

**Keywords:** antiseizure medication, attention deficit hyperactive disorders, autism spectrum disorders, Epilepsy, intellectual disability, neurodevelopmental disorders, Pregnancy, topiramate

## Abstract

**Introduction:**

Recent studies suggest an association between fetal topiramate exposure and an increased risk of neurodevelopmental disorders (NDD), such as autism spectrum disorders (ASD), intellectual disability (ID) and attention deficit hyperkinetic disorder (ADHD). This systematic review aimed to evaluate the evidence for such an association.

**Methods:**

A systematic literature search was conducted in Medline, Cochrane, and Embase on 16 December 2024, with automatic updates running until submission. Articles were examined if they included any number of offsprings of persons with epilepsy of childbearing potential (PWECP) exposed to topiramate during pregnancy. The main comparison was offspring of pregnant people without epilepsy and no antiseizure medication use, or alternatively antiseizure medication apart from topiramate. The search was limited to English language articles 2014–2024. The study followed PRISMA guidelines for reporting.

**Results:**

14 studies were included. The number of topiramate-exposed children ranged from 2 to 1,000. Most studies had a small group exposed to topiramate, and a large control group. Seven studies found a relationship between NDD outcomes and topiramate, three studies found no increased risk, and four studies were inconclusive. However, the risk seems mainly to be increased relative to no ASM use, and in many studies the risk was similar for lamotrigine and seemed lower than for valproate. Methodological approaches varied. Eleven studies were considered to be of good or fair quality, whereas three studies were considered to be of poor quality.

**Conclusion:**

This systematic review shows that topiramate prescribing requires caution in PWECP and that topiramate use during pregnancy warrants special attention to the neurodevelopment of the child. The risk seems to be increased relative to no ASM use, is not necessarily higher than the risk for lamotrigine, and lower than the risk of valproate.

## Introduction

Epilepsy is one of the most common neurological disorders, affecting more than 50 million individuals globally (WHO, 2024). People of all ages are affected, including people with epilepsy of childbearing potential (PWECP). Treatment usually involves prophylactic antiseizure medication (ASM) (WHO, 2024). Some ASMs pose risks for the developing child during pregnancy (Veroniki et al., 2017).

The ASM topiramate is effective against focal onset seizures, including those progressing to bilateral tonic-clonic seizures, as well as for generalized tonic-clonic seizures. However, topiramate use during pregnancy has come under scrutiny as several studies have found that pregnancy use is associated with low birth weight and with congenital malformations (Veroniki et al., 2017). Plausible biological mechanisms by which topiramate may affect fetal brain development include interference with neuronal migration, synaptogenesis, and neurotransmitter signaling during critical periods of neurodevelopment (Steele, 2024). In 2023, the Pharmacovigilance Risk Assessment Committee (PRAC) of the European Medicines Agency restricted the use of topiramate in PWECP and a pregnancy prevention program was recommended (EMA, 2023). This decision was based on evidence linking *in-utero* exposure to an increased risk of neurodevelopmental disorders (NDDs).

NDDs encompass several conditions caused by delayed or impaired maturing of the central nervous system. NDDs are characterized by impaired cognitive, emotional, or behavioral functioning, which typically presents during infancy or childhood as social challenges or communication difficulties (WHO, 2019). Autism spectrum disorders (ASD), intellectual disability (ID), and attention deficit hyperkinetic disorder (ADHD) represent the NDD diagnoses included in the 10th revision of International Classification of Diseases (ICD-10) (WHO, 2019). Some children with neurodevelopmental deficits do not completely fulfill the diagnostic criteria, while still having reduced daily living skills and communication difficulties (WHO, 2025).

While topiramate prescriptions to PWECP have been reduced with the new restrictions (Cerulli Irelli et al., 2024), it is still sometimes regarded as an attractive treatment alternative due to its effectiveness in epilepsy, and in other disorders such as migraine and obesity. All these disorders often occur in people of childbearing age with epilepsy. Assessing the safety profile of topiramate is essential for optimising care of PWECP and for giving precise information to users and potential users.

The aim of this study was to examine the risk of NDDs in the offspring when exposed to topiramate during fetal life.

## Methods

A systematic search was carried out on 16 December 2024, in The Cochrane Library, MEDLINE (Ovid), and EMBASE, with automatic updates running until submission. We considered original research articles, including preprints and conference abstracts, from the years 2014–2024, with automatic updates running until submission. The search was developed together with a trained librarian and based on the keywords “pregnancy”, “topiramate” and “epileptic disorder”, with synonyms and wildcard characters (Supplementary Tables 1-3). A snowball approach was employed by citation searching. In addition, three articles that did not appear in the search were included based on our previous acquaintance with the literature. Rayyan was used for literature sorting and duplicate removal (Ouzzani et al., 2016), and EndNote for reference management (Software, 2023).

Studies investigating NDDs in children of PWECP were included if any number of pregnancies involved topiramate exposure, either in monotherapy or polytherapy. We included all original studies including data on children of PWECP with perinatal topiramate exposure and NDDs, if available in English full text. However, we did not exclude studies that investigated topiramate-exposed children of mothers without epilepsy, even when results did not explicitly separate these populations. NDDs included ASD; ICD-10 F84. x, ADHD; ICD-10 F90. x and ID; ICD-10 F70-F79, as well as any psychometric instruments or proxy markers addressing any of these. Two first-authors (R.L and A.S) independently screened titles and abstracts, and read all fulltext articles. Quality appraisal was performed for all included studies using the Study Quality Assessment Tool for Observational Cohort and Cross-Sectional Studies by the National Institutes of Health (NHLBI, 2025). A third co-author (J.L) was involved to settle unresolved issues and disagreements. Studies were not excluded based on quality. Data extracted from the eligible studies were study design, source country, population, exposure including timing and dosage, comparator group, outcome definition, outcome measurement, key confounding variables, and key results. The Preferred Reporting Items for Systematic Reviews and Meta-Analyses (PRISMA) guidelines were followed for the search and study inclusion process (Page et al., 2021).

## Results

**FIGURE 1:**
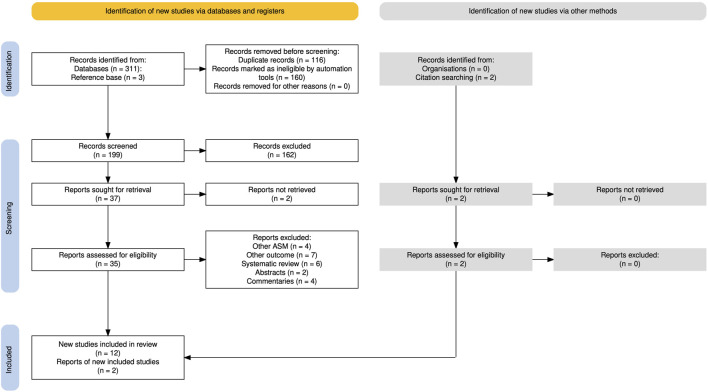
Inclusion-exclusion of studies, PRISMA flowchart (Haddaway et al., 2022).

A total of 199 citations were screened, of which 37 were retrieved in fulltext. After full-text review, we excluded 12 articles due to lack of original data, four due to absence of reported topiramate exposure, and seven articles that did not address neurodevelopmental outcomes. We included nine articles from the systematic search, supplemented by two articles identified through citation searching, and another three articles from our own reference base (Figure 1).

The 14 included studies comprised seven primary data collection studies (Table 1 and Supplement Table 5): four observational prospective studies (Rihtman et al., 2012; Nilsen Husebye et al., 2018; Nilsen Husebye et al., 2020; Meador et al., 2023), two cross-sectional studies (Bromley et al., 2016; Knight et al., 2023), and one retrospective study (Arkilo et al., 2015). Secondary data studies included five population-based registry studies (Bech et al., 2018; Blotière et al., 2020; Bjork et al., 2022; Dreier et al., 2023; Hernandez-Diaz et al., 2024; Madley-Dowd et al., 2024; Phillips et al., 2024), one claims-based cohort study (Hernandez-Diaz et al., 2024), and one pharmacovigilance study (Phillips et al., 2024) (Table 2 and Supplement Table 6).

**TABLE 1:** Primary data studies.

Study and study type	Country and years of inclusion	Total N	N children of PWECP	N children exposed to topiramate	Exposure definitions (timing, dosage)	Outcome measure	Outcome assessment time-window/Follow-up time	Number with the outcome among topiramate exposed	Comparison	Effect size measures	Quality appraisal: Poor (red) Fair (yellow) Good (green)	Our interpretation:^a^
Rihtman et al. (2012), observational prospective study	Israel 2001–2006	27	6	Mono: 9 (including 3 topiramate-exposed children of mothers without epilepsy)	All exposed (at least) during the first trimester. Dose in mg/day, mean (range): 208.33 (25–425)	The stanford-binet intelligence scales, 5th ed. (SB5)^b^ and use of special education	Preschool children between 3 years and 6 years 11month Mean age of the topiramate-group in months (range): 47.44 (36–68) Mean age of the control-group in months (range): 49.83 (37–68)	Special education school: 2 Education support in regular school: 1 Regular treatment in child development centers (occupational, physical or speech therapy): 5 (N children below a cut-off for the different scales not reported.)	Age- and gender-matched controls from the general population (1:2)	Z-values below -1.96: - VP: −2.565 - MC: −2.780 - GC: −2.104 - FM, M-FUN: −2.416 - GM, M-FUN: −2.019 - Fluid reasoning: −2.789 - Quantitative reasoning: −3.041 - Visual-spatial: −2.969 - VIQ: −2.394 - NVIQ: −2.550 - General IQ: −2.831	Poor	Results indicative of worse developmental outcomes for topiramate-exposed children. Limitations: Study group also included children of mothers without epilepsy and the sample size was small. Control group consisted of healthy controls
Arkilo et al. (2015), retrospective study	United States 2006–2011	62 (91 invitations, 24 excluded due to ASM polytherapy and 5 did not respond)	62	Mono: 2	Not reported	Abnormal developmental outcomes, defined as speech and/or delay requiring special services, as assessed by developmental specialists assigned by the school districts	Assessment at 2 years of age	0	No statistical comparisons performed	Not reported	Poor	Study did not find any abnormal developmental outcomes among the two topiramate exposed individuals. Limitation: Too small sample to conclude, no control group and no standardized validated outcome measures
Bromley et al. (2016), cross-sectional study	United Kingdom 2004–2007	185 (449 invitations, 45% participation rate)	185 (of which 55 not exposed to ASM	Mono: 27 (participation rate 53% for topiramate)	Dose around the time of conception (mg/day), mean (min-max): 271.3 (50–800)	Cognitive assessments conducted in the child’s home or school, blinded for authors^c^	Mean age (SD) at assessment, months: 76.7 (13.0)	N below average (<85): - Full-scale IQ: 3 - Verbal abilities: 3 - Nonverbal abilities: 3 - Processing speed: 3	1) Children of PWECP, no ASM 2) Comparisons across dose levels (0.5, 1.0, 1.5, and 2 times the median dose: 200 mg for topiramate). 3) Correlation at different dose levels to levetiracetam, valproate, or no ASM	1) And 2) topiramate better, or no statistically significant difference found.^d^ 3) No significant correlations for full scale IQ, verbal IQ or nonverbal IQ either to levetiracetam or no medication Valproate negatively correlated relative to topiramate on several dose levels and for several outcomes (i.e., valproate worse than topiramate)	Fair	Study suggests that topiramate-exposed children of PWECP have similar or better cognitive performance compared to children of PWECP exposed to other ASMs or to no ASMs. Limitation: Only 53% participation rate among topiramate-users, and 45% overall
Nilsen Husebye et al. (2018), observational prospective study	Norway 1999–2008	103,868	717 (of which 389 not exposed to ASM)	Mono: 19 Poly: 10	ASM-use self-reported in gestational week 17–19, validated to hospital reports. ASM-concentrations from blood samples collected gestational week 17–19	Global language delay and expressive language delay^e^	At age 18 and 36 months (expressive language delay only at age 36 months)	Mono: - Global language delay at 18 months: 2/11 - Global language delay 36 months: 0/8 - Expressive language delay 36 months: 1/6 (Polytherapy not reported separately for topiramate)	1) Children of PWECP exposed to any ASM 2) Children of PWECP not exposed to ASMs 3) Children of mothers without epilepsy 4) Dose-response relationship between topiramate-concentration and language scores	Effect estimates only reported with regards to periconceptional folate use yes/no. ORs here are calculated based on crude counts in Table 2 1) OR 0.93 2) OR 1.89 3) OR 1.71 4) No significant negative correlations (based on 7 and 4 maternal blood samples taken in gestational week 17–19, and 6 and 3 umbilical cord samples, correlated to language tests at age 18 months and age 36 months, respectively)	Fair	An effect of topiramate on language development cannot be ruled out. The study examined the effect of periconceptional use of folic acid supplementation, thus effect estimates were not reported for specific ASM-exposures. Limitation: Separate analyses for topiramate not reported. Small number of topiramate-exposed children
Nilsen Husebye et al. (2020), observational prospective study	Norway 1999–2008	114,408	734 (of which 388 not exposed to ASM)	Mono: 11 (of which 4 included with completed language questionnaire at 5 or 8 years) Poly: 9 (of which 4 included)	ASM-concentrations from blood samples collected gestational week 17–19	Language impairment^f^	At 5 years and 8 years	Mono: - Age 5: 2 - Age 8: 1 (Polytherapy not reported separately for topiramate.)	1) Children of mothers without epilepsy 2) Dose-response relationship between topiramate-concentration and language scores	1) 5 years: aOR 5.8 (0.5–64.0) 8 years: aOR 1.1 (0.1–10.9) 2) No significant negative correlations	Fair	Association between language impairment and topiramate-exposure cannot be ruled out. Limitations: Small number of topiramate-exposed children leading to wide confidence intervals. Only comparisons to children of mothers without epilepsy
Knight et al. (2023), cross-sectional study	United Kingdom 2019–2020	28	28 (of which 3 not exposed to ASM during pregnancy)	Mono: 25 (of which 21 included in main analysis. 4 excluded due to existing conditions known to effect neurodevelopment)	Mean dose 280 mg/day (range 100–800 mg/day) Low dose defined as ≤200. 23 throughout pregnancy. One discontinued at 6 weeks gestation	Based on VABS-III: Adaptive behavioural skills (ABC), including communication (COM), daily living (DLS) and socialisation skills (SOC) + Maternal reports from phone-interview^g^	Mean age 10.96 (range 2.58–17.33)	Number below the average (≤85) for - ABC: 9 - COM: 5 - DLS: 7 - SOC: 5 Maternal reports: - a) 5 - b) 6 - c) 3 - d) 6 - e) 4 (+2 among the children excluded due to pre-existing conditions)	1) VABS-III normative sample data (n = 2,560) 2) High versus low dose	1) Mean difference (95% CI): - ABC: 8.9 (-16.4, -1.37) - COM: −5.1 (−14.1, +3.9) - DLS: 9.6 (-15.6, -3.6) - SOC: 9.1 (-17.2, -1.1) 2) Negative correlations for ABC-, COM-, and SOC-scores	Fair	Study supports worse adaptive behaviour skills among topiramate-exposed children of PWECP compared to normative data as well as a dose-response relationship. Limitation: no comparison to other children of PWECP.
Meador et al. (2023), observational prospective study	United States 2012–2016	451	345	Mono: 3 Poly: <4 for each combination^g^	Max dose in 3rd trimester (mg/day). Median (min, Max): Mono (N = 3): 200 (150, 300) Poly (N = 3): 200 (200, 350) Max observed blood concentration in 3rd trimester (ug/mL), median (min, Max): Mono (N = 3): 6.4 (1.9, 7.8) Poly (N = 3): 2.5 (2.0, 3.7)	Primary outcome: Blindly assessed verbal index score. Secondary cognitive outcomes not reported for topiramate	At age 3 years	Verbal index score was measured for 2 children exposed to monotherapy topiramate, and imputed for one. (Polytherapy not reported separately for topiramate.)	Verbal index score mean (95% CI) in adjusted imputation analyses (Supplementary Table S11): - ASM-monotherapy overall: 103.2 (101.6, 104.9) - Lamotrigine: 101.9 (99.4, 104.4)	Main analysis not performed for topiramate-exposed due to low numbers (relationship of 3rd-trimester ASM-concentrations in children of PWECP). Crude graphical comparison to other specific ASMs show a lower verbal index score for topiramate monotherapy (Figure 3)	Good	Study suggests lower verbal scores for topiramate-exposed children. Limitation: Very small number of topiramate-exposed children

^a^ Black: indicates that topiramate increases the risk of NDDs. Grey: study inconclusive. White: study suggests no relationship between topiramate and NDDs, or a lower risk).

^b^ Visual-motor skills: visual motor integration (VMI), visual perception (VP) and motor coordination (MC) assessed by the Beery–Buktenica developmental test of visual-motor integration, fifth edition (Beery); Motor coordination problems: control during movement (CDM), fine motor (FM) and general coordination (GC) assessed by the Little developmental coordination disorder questionnaire (Little DCDQ); Functional motor skills: visual motor (VM), fine motor (FM), and gross motor (GM) skills, and participation based on a parent, a teacher and an assessor questionnaire of the Miller function and participation scales (M-FUN); Problem behaviors assessed by the Conners’ parent rating scale–revised: long form [CPRS-R] and Conners’ teacher rating scale–revised: long form [CTRS-R]; Intelligence and cognitive abilities including non-verbal IQ (NVIQ) and verbal IQ (VIQ).

^c^ Full-scale IQ (FSIQ), verbal index, nonverbal index, and processing speed index based on Wechsler Intelligence Scale for Children–Fourth Edition or the Wechsler Preschool and Primary Scale of Intelligence–Third Edition if the child was 5 years of age. Specific cognitive domains were assessed utilizing subtests from the NEPSY: A Developmental Neuropsychological Assessment, second edition and the Clinical Evaluation of Language Fundamentals–Fourth Edition (CELF), with parental rating of behavior collected using the Behavior Assessment System for Children, Second Edition (BASC).

^d^ 1) Auditory attention combined: 1.9 (0.4 to 3.4), indicating topiramate better. Inhibition–naming combined: 2.2 (0.6 to 3.7), indicating topiramate better. Theory of mind: 4.0 (1.2 to 7.6), indicating topiramate better. 2) Daily living: 5.7 (1.5 to 10.0), indicating topiramate better.

^e^ Global language delay measured by the communication scale from the Ages and Stages Questionnaires (ASQ) and expressive language delay defined as score 4 or less on the a one-item question on expressive language skills, where score 4 represents children talking in 2- to 3-word phrases, as reported by parents.

^f^ Defined as results outside cut-off for at least one of three parent-reported screening instruments: the communication scale from the Ages and Stages Questionnaires (ASQ), the Speech and Language Assessment Scale (SLAS), and the Norwegian instrument Twenty Statements about Language-related Difficulties (Language 20).

^g^ a) Did not meet early developmental milestones, b) Difficulties with learning at school, c) In receipt of formal supported learning provision, d) Difficulties with social interaction, e) Formal diagnosis of ASD.

^h^ Lacosamide + Topiramate, Lamotrigine + Topiramate, Levetiracetam + Topiramate, Lorazepam + Topiramate, Topiramate + Valproate, Felbamate + Oxcarbazepine + Topiramate.

Abbreviations: PWECP, people with epilepsy of childbearing potential; ASM, Anti-seizure medication; Mono = Monotherapy; Duo = Duotherapy; Poly = Polytherapy; OR, odds ratio; HR, hazard ratio; CI, confidence interval; IQR, interquartile range; NDD, neurodevelopmental disorder; ADHD, Attention-deficit hyperactivity disorder; ASD, Autism-spectrum disorder; ID, intellectual disability; ICD-10, the 10th revision of International Classification of Diseases.

**TABLE 2:** Secondary data studies.

Study and study type	Country and years of inclusion	Total N	N children of PWECP	N children exposed to Topiramate	Exposure definitions (timing, dosage)	Outcome measure	Outcome assessment time-window/Follow-up time	N with the outcome among topiramate exposed	Comparison	Effect size	Quality appraisal: Poor (red) Fair (yellow) Good (green)	Our interpretation:^a^
Bech et al. (2018),population-based registry study	Denmark 2005–2008	117,475	290(241/636 ASM-exposed pregnancies and 50/434 ASM-unexposed pregnancies)	Mono:17 (27 children of mothers with or without epilepsy)	Dispensing between 90 days prior to conception and birth. Subanalyses for trimester-specific exposure	Learning disability in the first year of compulsory education^b^	All children were assessed in the first-year o compulsory education (end of follow-up)	<4 (not restricted to children of PWECP)	1) Children without prenatal exposure to ASM (not restricted to PWECP) 2) Children with prenatal exposure to another ASM (not restricted to PWECP)	1) OR 5.82 (95% CI 1.21–27.97) 2) OR 2.57 (0.67–9.89)	Fair	Study indicates more learning disabilities among topiramate-exposed children compared to ASM-unexposed children. Compared to children exposed to other ASMs a similar number of learning disabilities could not be ruled out. Limitation: The observed effect could be due to indication bias
Blotiere et al. (2020), population-based registry study	France 2011–2014	1,721,990	Not reported. (9,034 children with prenatal exposure to any ASM-monotherapy were included, 2,916 exposed to lamotrigine)	Mono: 477 (children of PWECP or without epilepsy)^c^	At least one dispensing during pregnancy or within 30 days before	Any NDD (ICD-10 codes F70-F98), pervasive developmental disorders (PDD, F84), ID (F70–F79), or visits to aspeech therapist	Median follow-up 3.6 years (IQR 2.6–4.6)	Any NDD: 7 PDD: 1 ID: 2 Visits to speech therapist: 33 (not restricted to children of PWECP)	Children exposed to lamotrigine monotherapy (not restricted to PWECP)	Any NDD: HR 0.8 (0.4–1.9) PDD: HR 0.3 (0.0–4.9) ID: HR 0.5 (0.1–3.3) Speech therapist visit: HR 1.2 (0.8–1.8)	Good	Study did not rule out a similar risk of NDD in topiramate- and lamotrigine-exposed children. Limitation: Indication-stratified analyses not reported for topiramate due to low numbers
Bjørk et al. (2022), population-based registry study	Denmark 1997–2017, Finland 1996–2016, Iceland 2004–2017, Norway 2005–2017, Sweden 2006–2017	4,494,926	37,804(of which 21,634 not exposed to ASM)	Mono: 246(341 using extended exposure interval) Duo with lamotrigine: 123	1) ≥1 dispensing from lmp until birth. 2) Extended interval: ≥1 dispensing between 90 days before lmp and birth	At least one clinical diagnosis of ID, or ASD (ICD-10).^d^	Children’s mean age at diagnosis was between 6.1 and 7.9 years Median follow-up was 5.7 (IQR 2.9–9.1) years for topiramate-exposed	Mono: Any NDD: 10 Number of ASD cases, ID cases and NDD-cases from duotherapy were all non-zero but cannot be given owing to personal data protection restrictions	1) Children of PWECP, no ASM-exposure 2) Children of PWECP, no ASM-exposure. Extended exposure interval	1) Mono: ASD: HR 2.77 (1.35–5.65) ID: HR 3.47 (1.40–8.63) Any NDD: HR 2.13 (1.13–4.01) Duo with lamotrigine: Any NDD: HR 2.35 (1.13–4.87) 2) Mono: Any NDD: HR 1.75 (0.95–3.20) Duo with lamotrigine: Not reported	Good	Study indicates that topiramate-exposed children are at increased risk of NDD, including ASD and ID, compared to ASM-unexposed. Limitation: No comparison with children exposed to other ASMs. Relationship to specific exposure-windows during pregnancy was not addressed
Dreier et al. (2023), population-based registry study	Denmark 1997–2017, Finland 1996–2016, Iceland 2004–2017, Norway 2005–2017, Sweden 2006–2017	4,491,652	38,661(of which 22,203 not exposed to ASM)	Mono: 290	At least one dispensing during pregnancy or within 30 days before	A clinical diagnosis of ADHD, ASD, ID from specialist care	Children were diagnosed between 1 and 22 years of age. Mean follow-up 7 years for topiramate exposed	ASD:8 ADHD: 16 ID: 5	Children of PWECP, no ASM-exposure	ASD: HR 1.93 (0.95–3.94) ADHD: HR 2.38 (1.40–4.06) ID: HR 2.23 (0.90–5.50)	Good	Study indicates that topiramate-exposed children are at increased risk of NDD, compared to ASM-unexposed. Significant for ADHD and inconclusive for ASD and ID. Limitation: No comparison with children exposed to other ASMs. Dose-response relationship not addressed nor relationship to specific exposure-windows during pregnancy
Hernández-Díaz et al. (2024), claims-based cohort study	United States 2000–2020	4,212,121	28,952(of which 8,815 not exposed to ASM, 4,205 exposed to lamotrigine, and 800 exposed to valproate)	Mono: 1,030 (1,680 children based on early exposure of which 817 with early exposure only)	≥1 dispensing between gestational week 19 and delivery. (*Early exposure*: lmp through 18 weeks of gestation.) Median dose in late pregnancy200 mg/day, which was the cutoff for low/high dose	Clinical diagnosis of ASD (validated claims-based algoritm using ICD-10 codes)	Ages 1–18 years Median follow-up 2 years	17 (Including *early exposure*: 21 High dose: <11 Low dose: <11 Early and not late exposure: <11 Late and not early exposure: <11)	1) Children of PWECP, no ASM-exposure 2) Children of PWECP, lamotrigine-exposed	1) HR 0.96 (0.56–1.65) High-dose: HR 1.26 (0.57–2.78) Low-dose: HR 0.86 (0.44–1.69) Early exposure: HR 0.74 (0.44–1.24) Early and not late exposure: HR 0.53 (0.23–1.22) Late and not early exposure: HR 1.48 (0.54–4.05) 2) HR 1.22 (0.76–1.98)	Good	Study did not find an increased risk of ASD in topiramate-exposed children of PWECP compared to lamotrigine-exposed, or ASM-unexposed. However, an up to 65% increased risk cannot be ruled out Limitation: Small number of children with ASD and a limited number of chilren with long-term follow-up
Madley-Dowd et al. (2024), population-based registry study	United Kingdom 1995–2018, Sweden 1995–2020	United Kingdom: 518,047 Swe: 2,664,726	- United Kingdom: 6,508(of which 4,075 not exposed to ASM) - Swe: 17,785 (of which 10,769 not exposed to ASM)	Mono: - United Kingdom: 43 - Swe: 71 Poly: - United Kingdom: 57 (10.0%) - Swe: 157 (13.5%)	Supply of medicine in any pregnancy period based on prescription data (dispensing up to 30 days before lmp for swe)	Clinical diagnosis of ASD, ADHD, or ID by ICD-8/9/10 code, or prescription of ADHD medication (for swe)	Median follow-up in years: - United Kingdom: 7.51 (4.10–11.55) - Swe: 12.49 (6.49–19.27)	Mono: ASD: <5 (United Kingdom) + 6 (Swe) ADHD: 0 (United Kingdom) + <5 (Swe) ID: 0 (United Kingdom) + 5 (Swe) (Polytherapy results not reported separately for topiramate.)	Children of PWECP, no ASM-exposure	ASD: HR 2.30 (1.08–4.92) ADHD: HR 1.01 (0.38–2.71) ID: HR 5.73 (2.31–14.21) (Polytherapy results not reported separately for topiramate.)	Good	Study suggests an increased risk of NDD with topiramate exposure compared to no ASM exposure, significant for ASD and ID, and inconclusive for ADHD. Limitation: Relatively small number of outcomes. No comparison with children exposed to other ASMs. No dose-response relationships addressed or relationship to different exposure windows

Phillips et al. was not included in the table, since this study included no individual-level data and was based on spontaneous reporting data.

^a^ Black: indicates that topiramate increases the risk of NDDs. Grey: study inconclusive. White: study suggests no relationship between topiramate and NDDs, or a lower risk.

^b^ Defined either as a clinical diagnosis of intellectual disability (ICD-10: F7x), specific developmental disorders (F80–83), autism spectrum disorders (F84), emotional/behavioural disorders (F9x) or having special educational needs. Special educational needs included receiving special class lessons, additional time for a special education programme or having been classified with a learning disabilities obtained from the Special Education Register as part of the Danish Population Education Register.

^c^ < 100 children of PWECP, had prenatal exposure to ASMs, other than carbamazepine, levetiracetam, or valproic acid.

^d^ ASD, included codes for autism (F84.0), atypical autism (F84.1) and Asperger syndrome (F84.5). Any NDD, was defined as any of the above plus childhood disintegrative disorder (F84.3), disorder of intellectual disability and stereotyped movements (F84.4), unspecified pervasive developmental disorder (F84.8), or unspecified ID (F79).

^e^ Data standardized by total number of topiramate-prescriptions in the population: 4,975,166 topiramate-prescriptions. The overall number suspected adverse drug reactions related to topiramate: 206 per million topiramate-prescriptions, includes prescriptions to non-pregnant, non-epilepsy patients and men.

^f^ Including (our selection): Pregnancy related: a) exposure in pregnancy, b) foetal exposure during pregnancy, c) maternal exposure in pregnancy. Nervous system disorders: d) Neurological disorders not elsewhere classified (significant), e) Speech disorder, f) Speech disorder developmental, g) dyspraxia, h) dyslexia, i) mental impairment disorder. Psychiatric disorders: j) cognitive and attention disorders, k) learning disorders, l) ADHD, m) ASD, n) NDD.

Abbreviations:PWECP, people with epilepsy of childbearing potential; ASM, Anti-seizure medication; Mono = Monotherapy; Duo = Duotherapy; Poly = Polytherapy; OR, odds ratio; HR, hazard ratio; CI, confidence interval; IQR, interquartile range; NDD, neurodevelopmental disorder; ADHD, Attention-deficit hyperactivity disorder; ASD, Autism-spectrum disorder; ID, intellectual disability; ICD-10, the 10th revision of International Classification of Diseases.

The number of topiramate-exposed children ranged from two to about 1,000 in the included studies. Most studies aimed at investigating ASM-exposure broadly, and the number of topiramate-exposed children comprised only a small subset of the total study sample. Three primary data studies were primarily designed to examine topiramate exposure (Rihtman et al., 2012; Bromley et al., 2016; Knight et al., 2023) as were two secondary data studies (Bech et al., 2018; Hernandez-Diaz et al., 2024).

Most studies investigated only monotherapy, either by restricting the population at inclusion or during analyses.

One study investigated topiramate in monotherapy in main analysis but included duotherapy with lamotrigine in secondary analysis (Bjork et al., 2022). One study made no distinction between mono- and polytherapy (Phillips et al., 2024). Reported average daily doses of topiramate were generally around 200 mg.

### Primary data studies

Primary data studies collected new, original data directly from participants through tests, questionnaires and clinical assessments. Seven primary data studies were included (Rihtman et al., 2012; Arkilo et al., 2015; Bromley et al., 2016; Nilsen Husebye et al., 2018; Nilsen Husebye et al., 2020; Knight et al., 2023; Meador et al., 2023). Studies are presented below in chronological order.

A small study from 2012 (Rihtman et al., 2012) used a prospective design and validated psychometric tests to investigate the effect of prenatal topiramate monotherapy exposure in preschool children (3–6 years). The study included pregnant and pre-pregnant women that contacted the Israeli Teratogen Information Service concerning topiramate use. The study population consisted of six topiramate-exposed children of PWECP and three topiramate-exposed children of mothers with other indications for use. These were compared to children unexposed to topiramate, matched by age and gender. Three of the nine topiramate-exposed children received special education (33%) and five (56%) required occupational, physical, or speech therapy, while only two (11%) children of the control group received either. Topiramate-exposed children had significantly lower scores on several functional domains; including visual-motor, motor coordination, functional motor, and both verbal and non-verbal cognitive skills. Separate analyses were not conducted based on topiramate indication.

Two primary data studies found no associations between topiramate-exposure and NDDs during pregnancy (Arkilo et al., 2015; Bromley et al., 2016). One was a retrospective study including children of PWECP exposed to ASM in monotherapy (Arkilo et al., 2015). Of the 27 ASM-exposed children, only two were exposed to topiramate. At age 2 years none of these children had abnormal development as assessed by developmental specialists assigned by the school districts. This study did not include a comparison group, nor did they study dose-relationships. A cross-sectional study from the United Kingdom assessed 27 topiramate monotherapy exposed children of PWECP with full-scale IQ-tests and other validated cognitive scales (Bromley et al., 2016). When these were blindly compared to 55 children of PWECP unexposed to ASMs topiramate-exposed children performed similar on all tests, or even better. There was also no dose-relationship with the outcomes, and no significant difference between topiramate- and levetiracetam-exposure. Topiramate-exposed children performed better than the group exposed to valproate.

Another cross-sectional study from the United Kingdom employed the Vineland Adaptive Behaviour Scales (VABS-III), which measures adaptive behavioural skills, as well as communication and socialisation skills (Knight et al., 2023). These domains are associated with both ADHD, ASD, and ID. This study, which included 25 and assessed 21 topiramate monotherapy exposed children of PWECP, found reduced adaptive behaviour. This outcome showed a dose-response relationship. Six of the 25 topiramate-exposed children were formally diagnosed with ASD and nine of 21 had results below their predefined average range. However, the participation-rate was only 26% and no control cohort was included, hence results were compared to VABS-III normative data.

In the Norwegian Mother and Child Cohort (MoBa), language outcomes were prospectively assessed at ages 18 months, 36 months, 5 and 8 years (Nilsen Husebye et al., 2018; Nilsen Husebye et al., 2020). ASM-exposed children of PWECP had higher odds of language impairment than children of mothers without epilepsy (aOR 1.6 at 5 years and 2.0 at 8 years), but this was not statistically significant. Exposure to topiramate monotherapy was rare (N = 11, and only 4 with completed language questionnaires), resulting in unprecise estimates.

In the Maternal Outcomes and Neurodevelopmental Effects of Antiepileptic Drugs (MONEAD) study, a verbal index score was assessed at 3 years of age (Meador et al., 2023). This score was lower in children exposed to monotherapy topiramate compared to other ASM monotherapies. However, this was based on only two topiramate-exposed children, and three in imputed analyses. Comparative analyses were not performed separately for topiramate due to the small subsample size.

### Secondary data studies

A population-based case-cohort study employing Danish nationwide registry data 2005–2008 identified 27 children exposed to topiramate monotherapy, of which 17 of PWECP. This study found an increased risk of learning disability for topiramate-exposed children compared to children without ASM exposure (OR 5.8, 1.2–28.0) (Bech et al., 2018). However, the risk was not significantly increased compared to children exposed to other ASMs, and indication bias cannot be excluded, since children were not separated based on maternal indication of use.

The SCAN-AED studies combined nationwide health registry data from five Nordic countries. The first study included over 15,000 children of PWECP exposed to ASMs during pregnancy, including more than 250 children exposed to topiramate in monotherapy. This study found an increased risk of ASD (HR 2.8, 1.4–5.7) and ID (HR 3.5, 1.4–8.6) compared to children of PWECP unexposed to ASMs (Bjork et al., 2022). This was the only included study with specific results also for topiramate in polytherapy, showing that the risk of NDDs was increased also in children exposed to topiramate-lamotrigine duotherapy (HR 2.35, 1.13–4.87). The second paper from the SCAN-AED study, which included 290 children of PWECP exposed to topiramate in monotherapy, identified an increased risk of ADHD (HR 2.4, 1.4–4.1) when compared to ASM-unexposed children of PWECP (Dreier et al., 2023).

A registry-based cohort study from 2024, which combined data from Swedish national health registries and the Clinical Practice Research Datalink in the United Kingdom, demonstrated increased risks of ASD and ID associated with topiramate prescriptions in pregnancy (Madley-Dowd et al., 2024). The study included 418 children exposed to topiramate of which 114 were children of PWECP. Children of PWECP exposed to topiramate in monotherapy had an increased risk of ASD (HR 2.3, 95% CI: 1.1–4.9) and ID (HR 5.7, 2.3–14.2), but the risk of ADHD was not increased (HR 1.0, 0.4–2.7). In unstratified analyses, combining different indications of use, the risk of ID, but not ASD or ADHD, was increased.

In contrast, a US claims-based study from 2024 including 1,030 topiramate-exposed children found no association between topiramate-exposure and NDDs when analyses were restricted to PWECP (Hernandez-Diaz et al., 2024). Similarly, a French nationwide registry-based study from 2020, which included 477 topiramate-exposed children, found no effect on NDDs overall (HR 0.8, 0.4–1.9), intellectual disability (HR 0.5, 0.1–3.3), or pervasive developmental disorder (HR 0.3, 0.0–4.9) (Blotière et al., 2020). However, the study could not rule out if topiramate-exposed children were more likely to require speech therapy (HR 1.2, 0.8–1.8). In this study, separate indication-stratified analyses were reported for valproate, levetiracetam, carbamazepine and lamotrigine, but not for topiramate due to the small subsample size.

In 2024, a pharmacovigilance report which linked reports of suspected adverse drug reactions to prescription data from the English Open Prescribing database was published as a pre-print (Phillips et al., 2024). The study aimed to investigate pregnancy-related side effects of specific ASMs, including topiramate. The study found that topiramate, relative to other ASMs combined, had a higher number of reported reactions of nervous system disorders overall (45.6 per one million topiramate prescriptions, OR 1–2). This included 23 suspected cases of mental impairment disorder (4.6 per one million topiramate prescriptions). This study had limitations related to under-reporting of suspected adverse drug reactions. The quality of the exposure could not be ascertained (such as restriction to only perinatal exposures) and confounding was not addressed due to the lack of individual-level data.

## Discussion

This systematic review illustrates the complexity of assessing the relationship between *in utero* topiramate-exposure and NDDs. Several major reviews have previously investigated prenatal exposure to ASMs broadly and neurodevelopmental outcomes (Hope and Harris, 2023; Pack et al., 2024; Honybun et al., 2024). Recent EMA and MHRA pregnancy prevention measures for topiramate, together with discrepant emerging data, justify a focused review of topiramate specifically. Despite methodological differences among the 14 included studies, some consistent patterns emerged. Based on the included primary studies, topiramate-exposed children had lower visual perception, motor coordination, general coordination, fine- and gross motor skills, fluid- and quantitative reasoning, visuo-spatial skills, adaptive behavioural skills, and verbal IQ. There were more learning disabilities and NDD diagnoses among topiramate-exposed children, although three studies with good or fair quality came to the opposite conclusion. Inconsistent results were shown for full IQ. Common limitations across studies included a low number of topiramate-exposed children, confounding by indication not addressed, and lack of a standardized validated outcome measure.

To thoroughly assess causation, an important consideration is to establish a dose-response relationship (Hill, 1965). A dose-response was observed for adaptive behaviour (Knight et al., 2023), but no correlation was found for IQ (Bromley et al., 2016). High dose (≥100 mg/day) was related to a higher of NDDs in the SCAN-AED study (HR 2.93, 1.32–6.55) and low dose (<100 mg/day) was related to a less pronounced risk (1.71, 1.04–2.79), but these analyses were not performed in the epilepsy-restricted cohort (Bjork et al., 2022). Similarly, Hernandez et al. found that children exposed to high-dose (≥200 mg/day) had a slightly higher risk of ASD (HR 1.3, 0.6–2.8) than low-dose exposed (HR 0.9, 0.4–1.7), although statistical significance was not reached, and the overall conclusion of this study was negative (Hernandez-Diaz et al., 2024). Taken together, these findings suggest that the effect of topiramate is dose-dependent.

The registry-based studies had large sample sizes but variable attritions and lengths of follow-up. In the Nordic SCAN-AED studies the average age at NDD-diagnosis was 6.1–7.9 years (Bjork et al., 2022). Symptoms related to NDDs may become apparent only after a child has started in school, especially for ADHD or milder forms of ASD and ID. Insufficient observation time in the claims-based study from the US (Hernandez-Diaz et al., 2024) may explain why this study came to the opposite conclusion of the SCAN-AED studies, which found a 2-fold increased risk of ASD with topiramate exposure (Bjork et al., 2022). An alternative explanation for the discrepancy between these studies, is that the SCAN-AED studies did not use an active comparator, such as lamotrigine. The observed effect of topiramate in comparison to no ASM use could be due to unmeasured confounding by maternal epilepsy.

The registry-based study by Madley-Dowd et al. (2024) concluded with under-recording of NDDs in United Kingdom compared to Sweden, illustrating different country-specific recording practices and consequently potential misclassification bias. This is an inherent limitation of secondary data studies. Further, composite NDD outcomes were used to improve statistical power in the secondary data studies, but this may have obscured disorder-specific risk patterns. Primary data studies, on the other hand, collected new original data through both questionnaires and clinical assessment. However, studies that collected data from self-administered questionnaires, such as Husebye et al., or phone-interviews, such as Knight et al., may be affected by recall bias, which may overestimate the prevalence of the outcome. If this overestimation is non-differential, i.e., occurring just as much among exposed as unexposed, this should not impact the effect estimates. However, participation and response rates in primary data studies may have been differential, favoring participation both of children with NDDs and of exposed children. This may have skewed results away from the null. Some primary data studies used clinical assessments. While these assessments, when blinded, are the most objective outcome measures, their validity for estimating clinical NDDs have not been verified. The plurality of different scales also makes cross-study comparison challenging.

Madley-Dowd et al. found a higher risk of ID after restricting on indication, suggesting that not only maternal epilepsy contributes to the risk. Topiramate-exposed children were most often compared to children of PWECP unexposed to any ASMs, but some studies used an active comparator, lamotrigine or other ASMs, either in the epilepsy-restricted population (Bromley et al., 2016; Hernandez-Diaz et al., 2024) or in the overall population (Bech et al., 2018; Blotière et al., 2020; Phillips et al., 2024). Others compared topiramate-exposed children to ASM-unexposed children of mothers without epilepsy or to the general population (Rihtman et al., 2012; Bech et al., 2018; Nilsen Husebye et al., 2020; Knight et al., 2023). In these studies, potential bias by indication is highly relevant and limits the clinical value of the results.

Studies that rely on prescription data may be prone to exposure misclassification due to unobserved topiramate discontinuation during pregnancy. People with other indications for topiramate than epilepsy, such as migraine or weight-loss, are more prone to discontinuation during pregnancy (Cohen et al., 2020). One study showed that more than 75% of PWECP discontinued topiramate before the second trimester (Blotière et al., 2020). This study included topiramate-exposed children based on at least one dispensing event at any time during pregnancy or within 3 months before, without distinction of maternal indication. They observed no effect of topiramate on NDDs. This study obtained a large sample size but may have been impacted by exposure misclassification due to discontinuation around the time of conception.

Most studies defined exposure as first trimester exposure, whereas one secondary data study (Hernandez-Diaz et al., 2024), and two primary data studies (Nilsen Husebye et al., 2018; Meador et al., 2023), focused primarily on late-pregnancy exposure. They argued that this period is critical for synaptogenesis but found no association between ASD and late pregnancy topiramate exposure (HR 0.96, 0.56–1.65). It is essential that the exposure window matches the biologically sensitive development period with regards to the outcome. Failure to do so would bias the results towards the null. The Nordic health-registry studies, which relied on first-trimester exposure and found an effect of topiramate, (Bech et al., 2018; Bjork et al., 2022; Dreier et al., 2023; Madley-Dowd et al., 2024), may have underestimated this effect. Husebye et al. found that periconceptional folic acid use reduces the ASM-related risks (Nilsen Husebye et al., 2018). Although topiramate exposed group was small, these findings suggest that low folic acid levels may mediate the risk of NDD development associated with ASM use.

Only one study used sibling comparison to account for the unmeasured genetic and environmental confounding between siblings (Madley-Dowd et al., 2024). Several of the secondary data studies adjusted for maternal psychiatric disorders or maternal health, which may represent incomplete proxies of familial confounding (Bech et al., 2018; Blotière et al., 2020; Dreier et al., 2023; Bjork et al., 2022; Hernandez-Diaz et al., 2024). Knight accounted for family history of special educational needs, illnesses and neurodevelopmental conditions. Nilsen Husebye et al. (2020) adjusted for familial language impairment reported by the mother. In the MONEAD study, adjustments were made for both maternal and paternal IQ, and participants were excluded if maternal IQ was estimated to be below 70 (Meador et al., 2023). Bromley et al. (2016) also adjusted for maternal IQ. Residual confounding remains a possibility, particularly from unmeasured factors like genetic predispositions and other environmental exposures, including illegal drugs, psychiatric conditions of the mother, and paternal health factors. Such confounding is likely to bias estimates away from null. Registry-based studies were limited in regard to clinical details. Maternal cognitive function was not accounted for, which could have overestimated the effect of ASMs.

Strengths of this review included a systematic and comprehensive literature search, and an updated discussion including new studies on related and relevant topics. Several studies were not covered by earlier syntheses (Rihtman et al., 2012; Nilsen Husebye et al., 2018; Nilsen Husebye et al., 2020; Phillips et al., 2024; Madley-Dowd et al., 2024; Hernandez-Diaz et al., 2024). However, we did not include the PsycINFO database, and we did not check for new studies that may have been published after November 2025. Another limitation was the heterogeneity of study designs, which made it difficult to combine results, and we were unable to perform a meta-analysis. Although we adhered to a predefined PICO definition, the protocol was not prospectively registered.

In conclusion, topiramate use during pregnancy most probably increases the risk of ASD, ADHD and ID in a dose-dependent manner and independent of the mother’s underlying disease. The risk is obvious in contrast to no ASM medication, repeatedly shown in different epilepsy populations, but no difference to lamotrigine was found when directly compared. The low number of topiramate-exposed children, even in larger epilepsy-cohorts, has restricted direct comparisons of topiramate to other ASMs. However, the effect seems to be markedly lower than for valproate. Future research should focus on comparing the risks to other ASMs in monotherapy, as well as to relevant polytherapies. Such studies would further aid clinical decision-making, especially for patients where no ASM use is not a valid alternative. In future studies, extended follow-up and detailed information on drug use patterns in pregnancy, treatment indications, and other key covariates are important. Studies that account for shared genetic liability, such as primary data collection with genotype data or registry-based analyses using sibling designs, would also be informative.

## Data Availability

The original contributions presented in the study are included in the article/Supplementary Material, further inquiries can be directed to the corresponding author.
